# Mechanisms involved in drought stress tolerance triggered by rhizobia strains in wheat

**DOI:** 10.3389/fpls.2022.1036973

**Published:** 2022-11-10

**Authors:** Marcia Barquero, Jorge Poveda, Ana M. Laureano-Marín, Noemí Ortiz-Liébana, Javier Brañas, Fernando González-Andrés

**Affiliations:** ^1^ Institute of Environment, Natural Resources and Biodiversity, University of León, León, Spain; ^2^ Institute for Multidisciplinary Research in Applied Biology (IMAB), Universidad Pública de Navarra, Pamplona, Spain; ^3^ Centro de Tecnologías Agroambientales (CTA) Fertiberia - Edificio CITIUS (Centro de Investigación, Tecnología e Innovación) 1, Sevilla, Spain

**Keywords:** *Rhizobium*, drought, PGPR, abiotic stress, gene expression

## Abstract

*Rhizobium* spp. is a well-known microbial plant biostimulant in non-legume crops, but little is known about the mechanisms by which rhizobia enhance crop productivity under drought stress. This work analyzed the mechanisms involved in drought stress alleviation exerted by *Rhizobium leguminosarum* strains in wheat plants under water shortage conditions. Two (LBM1210 and LET4910) of the four *R*. *leguminosarum* strains significantly improved the growth parameters (fresh and dry aerial weight, FW and DW, respectively), chlorophyll content, and relative water content (RWC) compared to a non-inoculated control under water stress, providing values similar to or even higher for FW (+4%) and RWC (+2.3%) than the non-inoculated and non-stressed control. Some other biochemical parameters and gene expression explain the observed drought stress alleviation, namely the reduction of MDA, H_2_O_2_ (stronger when inoculating with LET4910), and ABA content (stronger when inoculating with LBM1210). In agreement with these results, inoculation with LET4910 downregulated *DREB2* and *CAT1* genes in plants under water deficiency and upregulated the *CYP707A1* gene, while inoculation with LBM1210 strongly upregulated the *CYP707A1* gene, which encodes an ABA catabolic enzyme. Conversely, from our results, ethylene metabolism did not seem to be involved in the alleviation of drought stress exerted by the two strains, as the expression of the *CTR1* gene was very similar in all treatments and controls. The obtained results regarding the effect of the analyzed strains in alleviating drought stress are very relevant in the present situation of climate change, which negatively influences agricultural production.

## 1 Introduction

Wheat (*Triticum aestivum* L.) is the world’s leading cereal crop, being the main ingredient of the human diet in many parts of the world (Arshad, 2021), with 219 million ha cultivated in 2020 worldwide, producing 761 million tons of grain Food and Agriculture Organization of the United Nations (FAO) (2022). In the current climate change scenario, it has been demonstrated that the main problems that affect wheat productivity in the world are high temperatures and drought, making the development of heat- and drought-resistant high-yielding varieties necessary to ensure food security (Ali et al., 2017).

Drought is considered the most far-reaching of all natural disasters, affecting natural ecosystems and numerous human activities, such as agriculture, water access, energy, tourism, and basic human welfare (Haile et al., 2020). However, agriculture is the human sector most affected by climate change, as it is directly dependent on rainfall and evapotranspiration, and water shortages cause a decrease in crop yield and quality (Parsons et al., 2019). It is estimated that the yield reduction caused by a dry year can range between 1–20% (Musolino et al., 2018); in the specific case of wheat, a meta-analysis of 55 publications concluded that drought causes an average 27% decrease in yield (Zhang et al., 2018). Despite an estimated doubling of water demand for agriculture by 2050, freshwater availability is projected to decrease by 50% due to climate change (Gupta et al., 2020). Of particular concern are the effects that global climate change can produce in the agriculture of specific regions more prone to be affected by the drought intensification, as e.g. southern Europe and northern and southern Africa (Meza et al., 2020).

Drought causes different morphological, physiological, and biochemical changes related to abiotic stress in plants (Seleiman et al., 2021). To cope with drought situations, plants have developed different mechanisms, such as increased root water uptake, reduction in water loss by closing stomata, and activation of hormonal responses (mainly mediated by abscisic acid [ABA]), leading to the production of specific metabolites and increased antioxidant activity (Gupta et al., 2020). These mechanisms have been studied extensively in wheat (Sallam et al., 2019).

The main strategy for preventing and combating the detrimental effects of drought on crops has been the use and development of tolerant varieties (Kapoor et al., 2020; Rosero et al., 2020). There have been some attempts at the exogenous application of growth regulators, osmoprotectants, and plant mineral nutrients, but the response obtained in real field conditions has been limited and erratic (Marthandan et al., 2020; Seleiman et al., 2021). Recent research has shown that the main modulator of plant tolerance to drought is the rhizosphere and the microbiome associated with it (Zia et al., 2021). Thus, the main advances in increasing drought tolerance in the field are being achieved in the rhizosphere, e.g., adding beneficial microorganisms, hydrogels, nanoparticles (Seleiman et al., 2021; Zia et al., 2021), and seed priming (Marthandan et al., 2020).

Microbial plant biostimulants (MPBs) can alleviate the effects of drought on crops (Poveda, 2021a). MPBs effective for crops under drought conditions consist of plant growth-promoting bacteria (PGPB), including cyanobacteria, and plant growth-promoting fungi (PGPF), notably mycorrhizal fungi (Del Buono, 2021). The mechanisms of action by which rhizobacteria improve plant tolerance to drought involve the modification of phytohormone activity (Vurukonda et al., 2016; Poveda and González-Andrés, 2021), antioxidant defenses in plant tissues (Ngumbi and Kloepper, 2016; Vurukonda et al., 2016), the production of microbial volatile organic compounds (mVOCs) (Poveda, 2021b), 1-aminocyclopropane-1-carboxylate (ACC) deaminase (Ojuederie et al., 2019), osmolytes, and/or exopolysaccharides (Kaushal and Wani, 2016). In wheat, the main rhizobacterial genera described as MPBs under drought conditions include *Azospirillum*, *Bacillus*, *Paenibacillus*, and *Pseudomonas* (Verma and Suman, 2018).

The genus *Rhizobium* is well known for its ability to form symbioses with legumes and fix atmospheric nitrogen in root nodules (Naseer et al., 2019). In addition, rhizobia species can act as PGPB through other mechanisms, such as phosphorus solubilization and the production of siderophores, phytohormones, exopolysaccharides, enzymes, and secondary metabolites (Naseer et al., 2019). Interestingly, the plant growth-promoting effect of rhizobia can also be exploited in non-leguminous crops (Díez-Méndez and Menéndez, 2021), including cereals (Goyal et al., 2021). However, the effect of *Rhizobium* species to alleviate drought situations in cereals has not yet been studied in depth; although in legumes, it has been reported to increase plant tolerance to stress through mechanisms of action such as the production of osmolytes, mVOCs, phytohormones, and ACC deaminase and nutrient uptake (Singh et al., 2021).

The objective of this work was to evaluate the action of rhizobia strains in conferring drought tolerance to inoculated wheat plants that are exposed to reduced availability of water. Likewise, this work attempts to ascertain the mechanisms of action of rhizobia as PGPB involved in improving the tolerance of crops to water shortage situations through the study of growth, physiological, and biochemical parameters, as well as gene expression patterns in wheat plants.

## 2 Materials and methods

### 2.1 Strain description and growth conditions

The strains used in this study, namely LCS2403, LBM1210, LET4910, and LPZ2704 (hereafter LCS, LBM, LET, and LPZ, respectively), belong to the species *Rhizobium leguminosarum* and have been patented in combination with a complex mineral fertilizer to be used in the fertilization of cereals and horticultural crops (Mulas et al., 2018). They present the following relevant PGP properties (Mulas, 2015): LCS, IAA production (11.2 µg IAA 10^−8^ CFU); LBM, solubilization of Ca_3_(PO_4_)_2_ in Petri dishes; LET, IAA production (11.6 µg IAA 10^−8^ CFU) and siderophore production observed as orange halo in M9-CAS agar medium modified; and LPZ, ACC deaminase activity (435.25 µmol ACC mg prot^−1^ h^−1^).

### 2.2 Plant drought stress assay

*Rhizobium* strains LCS2403, LBM1210, LET4910, and LPZ2704 were used to inoculate the wheat ‘Galera-R2’ variety. The assay was established in a growth chamber under controlled conditions at 25°C for 10 h in light and 14°C for 14 h in darkness. Wheat seeds were surface sterilized by soaking for 1 min in 70% (v/v) ethanol and for 5 min in 2% (v/v) sodium hypochlorite, then rinsed three times with sterile distilled water and germinated in the dark on water agar plates (15 g/l).

After 48 hours, germinated seeds were planted in 1 L pots (5 seeds per pot) using a mixture of washed sterile vermiculite and sterile soil (1:1) as a substrate. The experimental design was a complete randomized design consisting of 11 pots with 5 plants each per treatment (55 plants per treatment). There were 10 treatments, including controls: control with water stress conditions and without bacterial inoculation (W-); positive control: without water deficiency and without bacterial inoculation (W+); four treatments with water stress conditions and inoculated with one of the rhizobia strains (LBM-W, LCS-W, LET-W, and LPZ-W); and four treatments without water deficiency and inoculated with one of the rhizobia strains (LBM1210+W, LCS0403+W, LET1910+W, and LPZ0704+W). In treatments inoculated with rhizobia strains, 200 µl of a suspension of 10^9^ CFU ml^−1^ obtained after incubation at 28°C in YMB for a period of 5 days was added to each seed. CFU were estimated by plating 10-fold serial dilutions on YMA plates. Plants were grown for 25 days.

Pots with seeds were irrigated and sown with 200 ml of distilled water. Four days after sowing, pots were watered with a half-strength Hoagland solution: 0.5 M Ca(NO_3_)_2_; 0.5 M KNO_3_; 0.5 M MgSO_4_·7H_2_O; 0.5 M KH_2_PO_4_; 20 mg/l FeEDTA; 0.5 M CaCl_2_; 1.43 g/l H_3_BO_3_; 0.905 g/l MnCl_2_ 4H_2_O; 0.055 g/l ZnCl_2_; 0.025 g/l CuSO_4_·5H_2_O; and 0.0125 g/l Na_2_MoO_4_·2H_2_O. All irrigations were done from a bottom reservoir to avoid rhizobia strain loss by washing.

Water-stressed treatments were not watered until the end of the experiment. The remaining treatments were watered every 7 days, alternating between half-strength Hoagland solution and distilled water until the end of the experiment. All treatments—water stressed and non-water stressed—received a re-inoculation with the rhizobia stains 7 days before the end of the experiment with 1 ml per plant of a suspension of 10^9^ CFU ml^−1^.

At the end of the experiment, the chlorophyll content of five replicates was determined with a SPAD chlorophyll meter (SPAD–502 Minolta Co. Ltd., Osaka, Japan), measuring the last completely developed leaf of the wheat plant and obtaining SPAD values. Five replicates were collected. Fresh (FW) and dry (DW) aerial weights were determined. Aerial biomass was dried in an oven at 70°C for 48 h to obtain dry biomass values. Appropriate analysis of variance was performed using the statistical package SPSS v. 17.0. Additionally, the relative water content (RWC) of the aerial biomass was estimated according to the formula RWC (%) = [(FW−DW)/FW]*100 (Zhang and Blumwald, 2001).

Plants of the remaining six replicates were harvested, immediately frozen in liquid nitrogen, and stored at −80°C for further biochemical and molecular analyses.

### 2.3 Biochemical analysis

Based on the results obtained in the plant drought stress assay, three replicates per treatment (each replicate composed of five plants) were used for biochemical analysis. The malondialdehyde (MDA), hydrogen peroxide (H_2_O_2_), proline, and abscisic acid (ABA) content were determined. An analysis of variance was performed using the statistical package SPSS v. 17.0.

#### 2.3.1 Lipid Peroxidation (MDA content)

Lipid peroxidation in leaves was measured with the thiobarbituric acid (TBA) test following Heath and Packer (1968), based on MDA content, as described in Velikova et al. (2000). The TBA test determines MDA as an end-product of lipid peroxidation. Leaves (500 mg) were homogenized in 5 ml 0.1% (w/v) trichloracetic acid (TCA) solution and then centrifuged at 10,000×g for 20 min. The supernatant (0.5 ml) was added to 1 ml 0.5% (w/v) TBA in 20% TCA. The mixture was incubated in boiling water for 30 min and then placed in an ice bath to stop the reaction, following centrifugation at 10,000×g for 5 min. The absorbance of the supernatant was read at 532 nm. The value for non-specific absorption at 600 nm was subtracted. The amount of MDA–TBA complex (red pigment) was calculated from the extinction coefficient 155 mM^−1^ cm^−1^, as follows: MDA (nmol/ml FW)=[(A_532nm_−A_600nm_)/155000]x1000000.

#### 2.3.2 Hydrogen peroxide content

Leaf tissues (500 mg) were homogenized in an ice bath with 5 ml of 0.1% (w/v) TCA. The homogenate was centrifuged at 12,000×g for 15 min, and 0.5 ml of the supernatant was added to 0.5 ml 10 mM potassium phosphate buffer (pH 7.0) and 1 ml 1 M KI. The absorbance of the supernatant was read at 390 nm. The H_2_O_2_ content was determined using a standard curve (Velikova et al., 2000).

#### 2.3.3 Proline content

Proline content was analyzed using an ethanolic extract prepared by homogenizing 100 mg of fresh tissue in 1 ml of 70% ethanol (Tiwari et al., 2017). The reaction mixture constituted 1% (w/v) ninhydrin in 60% (v/v) acetic acid and 20% (v/v) ethanol, mixed with ethanolic extract in a ratio of 2:1. The 100 µl reaction mixture was then incubated in a water bath at 95°C for 20 min and cooled to room temperature. Absorbance was recorded at 520 nm in a microplate reader.

#### 2.3.4 ABA content

The ABA content was quantified with the Phytodetek-ABA Immunoassay kit (Agdia, Elkhart, IN), following kit specifications. Sample extraction was carried out as described by Barnawal et al. (2017). Leaf samples were ground with liquid nitrogen, and 0.5 g of ground samples were suspended in 8 ml of extraction solution (80% methanol, 100 ml l^−1^ butylated hydroxytoluene (BHT), and 0.5 g l^−1^ citric acid monohydrate) and agitated overnight at 4°C in the dark. The extraction solution was centrifuged at 1000×g for 20 min at 4°C. The supernatant was transferred to a new tube and dried in a vacuum. The dry supernatant was dissolved in 100 µl of 100% methanol and 900 µl of Tris-buffered saline [TBS (pH 7.8)] and was used in the Phytodetek-ABA Immunoassay kit.

### 2.4 Quantitative reverse transcription-polymerase chain reaction analysis of drought stress-related genes in wheat plants

The analysis of drought stress-related genes in wheat plants inoculated and non-inoculated with rhizobia strains exposed to water stress was carried out to explore the physiological mechanisms involved in wheat drought tolerance. The analyzed genes were *DREB2*, *CAT1*, *CTR1*, and *CYP707A1* (**Table 1**). Based on the results obtained in the plant drought stress assay, three replicates per treatment (each replicate composed of five plants) were analyzed. RNA extraction was done with TRIzol reagent (Invitrogen, Waltham, Massachusetts, USA) and cleaned up with the Direct-zol RNA Miniprep Kit (Zymo Research, Irvine, CA, USA), including DNase enzymatic treatment. The RNA quality and quantity were determined with the Invitrogen™ Qubit™ 4 Fluorometer, and RNA integrity was confirmed by agarose gel electrophoresis. cDNA synthesis was performed using 2 μg mRNA in the Just cDNA™ Double-Stranded cDNA Synthesis Kit (Stratagene, La Jolla, CA, USA), according to the manufacturer’s instructions. qRT-PCR was performed using an Agilent Mx3005P Real-Time PCR System (Agilent Technologies, Santa Clara, CA, USA) with Luna ^®^ Universal qPCR Mastermix (New England Biolabs, Ipswich, MA, USA). All PCR reactions were performed in triplicate in a total volume of 10 μL for 40 cycles under the following conditions: denaturation at 95°C for 15 s and annealing and extension at 60°C for 30 s. The melting curve was built according to real-time instrument recommendations. Threshold cycles (CT) were calculated using the wheat *Actin* gene (**Table 1**) as an endogenous control. Data were analyzed using the 2^−ΔΔCT^ method described by (Livak and Schmittgen, 2001). Real-time PCR Miner algorithm was used to determine the efficiency of each PCR reaction; the algorithm analyzes real-time PCR data from raw fluorescence data (Zhao and Fernald, 2005). The independent samples *t-*test was used to determine significant differences in relative gene expression (SPSS v. 17.0).

**Table 1:** List of genes and primers used in qRT-PCR.

Gene	Primer sequence	Functional annotation	Reference
*DREB2*	DREB2-F: 5´-CGGAGATGCAGCTTCTTGATT-3´ DREB2-R: 5´-GATCTCGAGCG ACGGGTACTT-3´	Encoding for an abiotic stress-responsive transcription factor: dehydration responsive element binding protein 2 in wheat	(Barnawal et al., 2017)
*CAT1*	CAT1-F: 5´-CCATCTGGCTCTCCTACTGG-3´ CAT1-R: 5´-AGAACTTGGACGGCCCTGA-3´	Encoding for catalase, an enzyme essential for elimination of H_2_O_2_ produced through photorespiration under stress conditions	(Gontia-Mishra et al., 2016)
*CTR1*	CTR1-F: 5´ -GCTGCTCTTGTTGAATCCTGTTG-3´ CTR1-R: 5´-ATCCACAATGCTTGAAAACGAA-3´	Encoding for a regulatory component of the ethylene signalling pathway that modulates stress related changes in plants	(Barnawal et al., 2017)
*CYP707A1*	CY-F: 5´-GCGCACCTCTTCAAGCCTA-3´ CY-R: 5´-CGAAGATGGACAGCAATGC-3´	Encoding for ABA catabolic enzyme, ABA8`-hydroxylase, controlling ABA content in the plant tissue	(Han et al., 2019)
Actin	Act-F: 5´-CGAAACCTTCAGTTGCCCAGCAAT-3´ Act-R: 5´-ACCATCACCAGAGTCGAGCACAAT-3´	Constitutive gen as endogenous contol.	(Barnawal et al., 2017)

### 2.5 PCA analysis

The means of the plant growth, physiological, and biochemical parameters and gene expression results were analyzed using principal component analysis (PCA) to visualize the clustering of treatments. PCA analysis was carried out with PRIMER v7 (Clarke and Gorley, 2015).

## 3 Results

### 3.1 Plant drought stress assay

**Figure 1:**
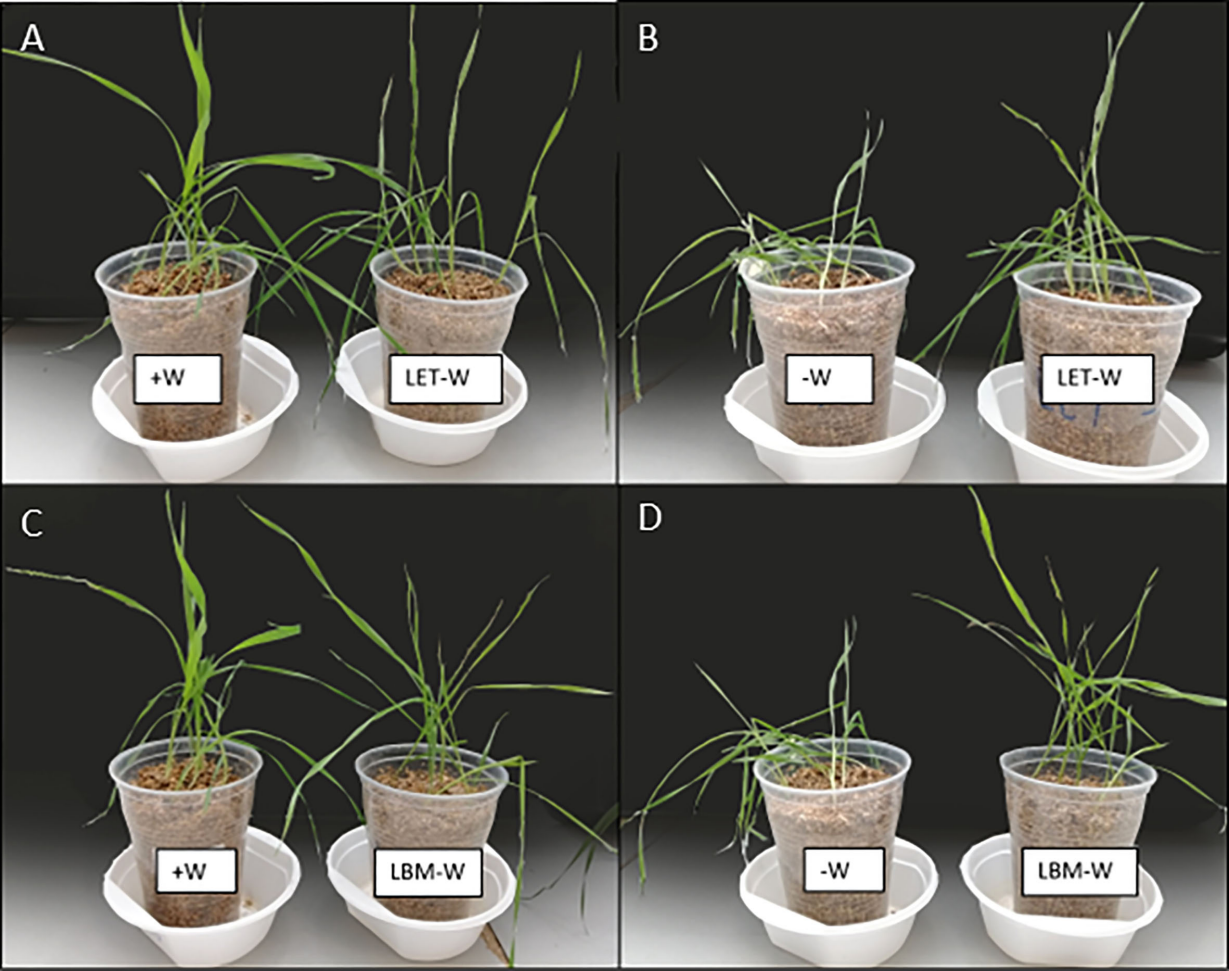
Effect of rhizobia strains LET and LBM inoculations on wheat plants under water-stress conditions compared to non-stressed wheat plants **(A, C)** and water-stressed wheat plants without rhizobia inoculations **(B, D)**.

**Figure 2:**
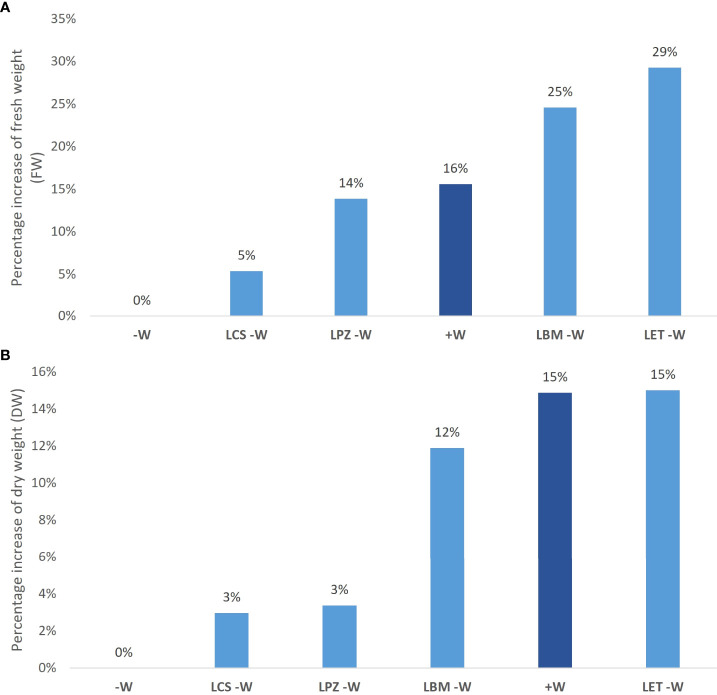
Effect of rhizobia strain inoculations on wheat plants under water-stress conditions. Percentage increase in aerial fresh weight (FW) **(A)** and aerial dry weight **(B)** of wheat plants inoculated with rhizobia strains LBM, LCS, LET, and LPZ under water-stress conditions and non-water stressed/non-inoculated plants (+W) compared to water-stress/non-inoculated wheat plants (-W).

The inoculation of water-stressed wheat plants improved drought stress tolerance. Plants under water deficiency and inoculated with bacteria showed increased FW and DW compared to non-inoculated plants (-W) (**Table 2**; **Figures 1**, **2**). The increase in FW was significant for all bacterial strains except for LCS (**Table 2**), being as high as 25% for LBM and 29% for LET, while the increase in DW was significant in plants inoculated with LMB (12% increase) and LET (15% increase). Interestingly, the FW of plants under water deficiency inoculated with LBM and LET was even higher than in the non-inoculated controls without water deficiency (+W) (**Figure 2**). Moreover, the inoculation of non-stressed plants with the bacterial strains promoted plant growth, increasing the FW by 23% (LCS), 35% (LET), 62% (LPZ), and 80% (LBM), and the DW by 3% (LET), 13% (LPZ; LCS), and 21% (LBM) (**Table 2**). In agreement with the results obtained for FW and DW, inoculation also improved the water content of water-stressed plants; therefore, the RWC was significantly higher in plants inoculated with all the strains except for LCS, while the highest improvement in RWC was recorded in plants inoculated with LBM and LET (**Table 2**). Moreover, SPAD reached the highest values in plants under water deficiency and inoculated with LBM and LET, along with the non-stressed plants (+W), although the differences were not significant between the stressed and non-inoculated plants.

**Table 2:** Mean values and standard errors of aerial fresh and dry weight, RWC and SPAD values of wheat plants grown under drought stress vrs non-stress.

Treatment	Aerial fresh weight (mg)	Aerial dry weight (mg)	Water content [RWC] (%)	SPAD values
-W	1628,0 ^g^ ± 71,2	295,8 ^f^ ± 6,5	81,8 ^d^ ± 1,0	37,7 ^b^ ± 1,0
+W	1882,0 ^f^ ± 44,4	339,8 ^cd^ ± 11,9	81,9 ^cd^ ± 0,8	41,1 ^a^ ± 0,5
LBM -W	2028,2 ^e^ ± 73,9	331,0 ^cde^ ± 5,5	83,7 ^b^ ± 0,5	41,1 ^a^ ± 0,8
LBM +W	3382,6 ^a^ ± 61,5	412,0 ^a^ ± 23,6	87,8 ^a^ ± 0,7	39,8 ^a^ ± 0,8
LCS -W	1716,0 ^g^ ± 52,2	304,6 ^ef^ ± 5,4	82,2 ^bcd^ ± 0,4	39,9 ^a^ ± 0,8
LCS +W	2320,6 ^d^ ± 42,8	384,6 ^a^ ± 22,5	83,4 ^bc^ ± 0,9	40,6 ^a^ ± 0,9
LET -W	2104,8 ^e^ ± 64,0	340,2 ^cd^ ± 11,8	83,8 ^b^ ± 0,6	40,7 ^a^ ± 0,8
LET +W	2548,0 ^c^ ± 65,7	349,0 ^bc^ ± 21,4	86,3 ^a^ ± 0,9	40,0 ^a^ ± 1,8
LPZ -W	1854,0 ^f^ ± 42,8	305,8 ^def^ ± 18,7	83,5 ^bc^ ± 0,9	40,2 ^a^ ± 1,1
LPZ +W	3056,0 ^b^ ± 61,9	382,6 ^ab^ ± 19,6	87,5 ^a^ ± 0,5	40,5 ^a^ ± 0,6
ANOVA Mean Square	1700402,6	7400,2	24,7	4,9
F value	486,866	28,029	41,798	5,281
Significance	***	***	***	***

(Significance level: *** p ≤ 0.001). A Tukey’s test was used to compare mean values; the means followed by the same letter did not significantly differ for p ≤ 0.05.

Growth (aerial FW and DW) and physiological parameters (RWC and SPAD) showed that the most significant effect in improving drought stress tolerance was exerted by the bacterial strains LBM and LET. Therefore, biochemical and gene expression analyses were carried out in stressed plants inoculated with the above-mentioned strains.

### 3.2 Biochemical analysis

The analysis of MDA, H_2_O_2_, proline and ABA content in wheat plants supports the results obtained in growth and physiological parameters, reinforcing the role of LBM and LET in conferring drought stress tolerance.

**Figure 3:**
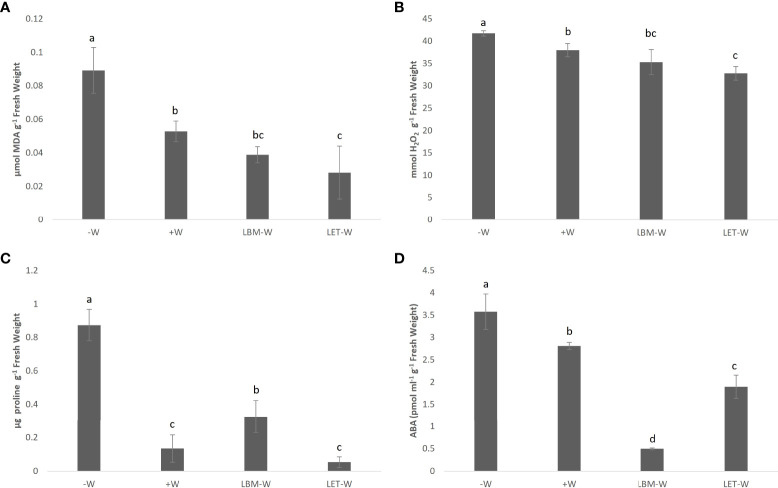
Effect of rhizobia strains LBM and LET inoculations on **(A)** MDA, **(B)** H_2_O_2_ **(C)** proline, and **(D)** ABA content of wheat plants under water-stress conditions (LBM-W and LET-W). Water-stress/non-inoculated wheat plants (-W) and non-water stress/non-inoculated plants (+W). Controls were included in the analysis. Mean values and standard deviation of three replicates. Tukey’s test was used to compare mean values; means followed by the same letter did not significantly differ at p ≤ 0.05.

The MDA and H_2_O_2_ content in plant tissues decreased significantly when plants under water deficiency were inoculated with bacteria. The level of these compounds was even lower than in non-stressed plants, and this difference was significant in plants inoculated with LET (**Figures 3A, B**).

Proline levels in non-inoculated and water-stressed plants were significantly higher than in inoculated plants and in the W+ control. Similarly, for MDA and H_2_O_2_, the lowest value was obtained in plants under water deficiency and inoculated with LET. Although this value did not differ statistically from the control W+, it was numerically lower (**Figure 3C**). Otherwise, the stressed plants inoculated with LBM showed a higher proline content than the positive control.

Similar to the results described for the other biochemical parameters, ABA accumulation was significantly lower in plants under water deficiency inoculated with rhizobia than in the stressed control (-W) and in the positive control (+W). Interestingly, in contrast to the other parameters, LBM instead of LET produced the sharpest decline in ABA accumulation (**Figure 3D**).

### 3.3 Analysis of drought stress-related genes in wheat plants

The relative expression of drought stress-related genes in the positive control (+W) and in the inoculated treatments under water deficiency was compared with the expression in the water-stressed/non-inoculated control (-W), which was used as the reference. Different patterns were observed.

**Figure 4:**
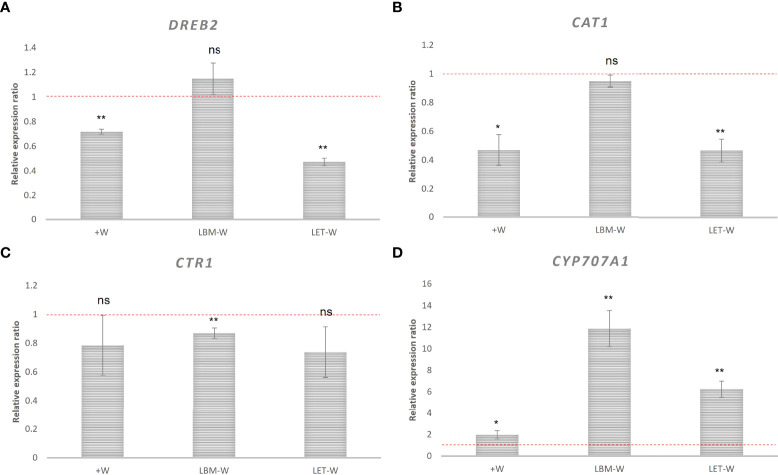
Relative expression of wheat genes related to drought stress: **(A)** *DREB2*, **(B)** *CAT1*, **(C)** *CTR1*, and **(D)** *CYP707A1*. Relative expression ratio of wheat plants inoculated with rhizobia strains LBM and LET under water-stress conditions (LBM-W) (LET-W) and non-water stress/non-inoculated plants (+W) with respect to water-stress/non-inoculated wheat plants (-W). Data were analyzed using 2^−ΔΔCT^ method described by Pfaffl (2001). Mean values and standard deviations of the relative expression of three biological replicates. The independent samples *t*-test was used to determine significant differences in relative gene expression (significance level: ** 0.001 < p ≤ 0.01; * 0.01 < p ≤ 0.05; ns, not significant). Values below the red dashed line are downregulated.

*DREB2* was downregulated in the positive control (+W) and in the treatment inoculated with the LET strain but not in the treatment inoculated with LBM, whose expression did not significantly differ from the expression in -W (**Figure 4A**).

The *CAT1* gene was also downregulated: 2 fold in the control +W and in the treatment inoculated with LET, and 1 fold in the treatment inoculated with LBM, although in plants inoculated with LBM, there was no statistical significance (**Figure 4B**). Expression dynamics of *CTR1* gene was similar to *CAT1*, but unlike this, only the down-regulation in plants inoculated with LBM was significant (**Figure 4C**). In contrast, *CYPP707A1* was upregulated in both +W and in the treatments inoculated with rhizobia (**Figure 4D**).

### 3.4 Relationship between the analyzed parameters explained by PCA

**Figure 5:**
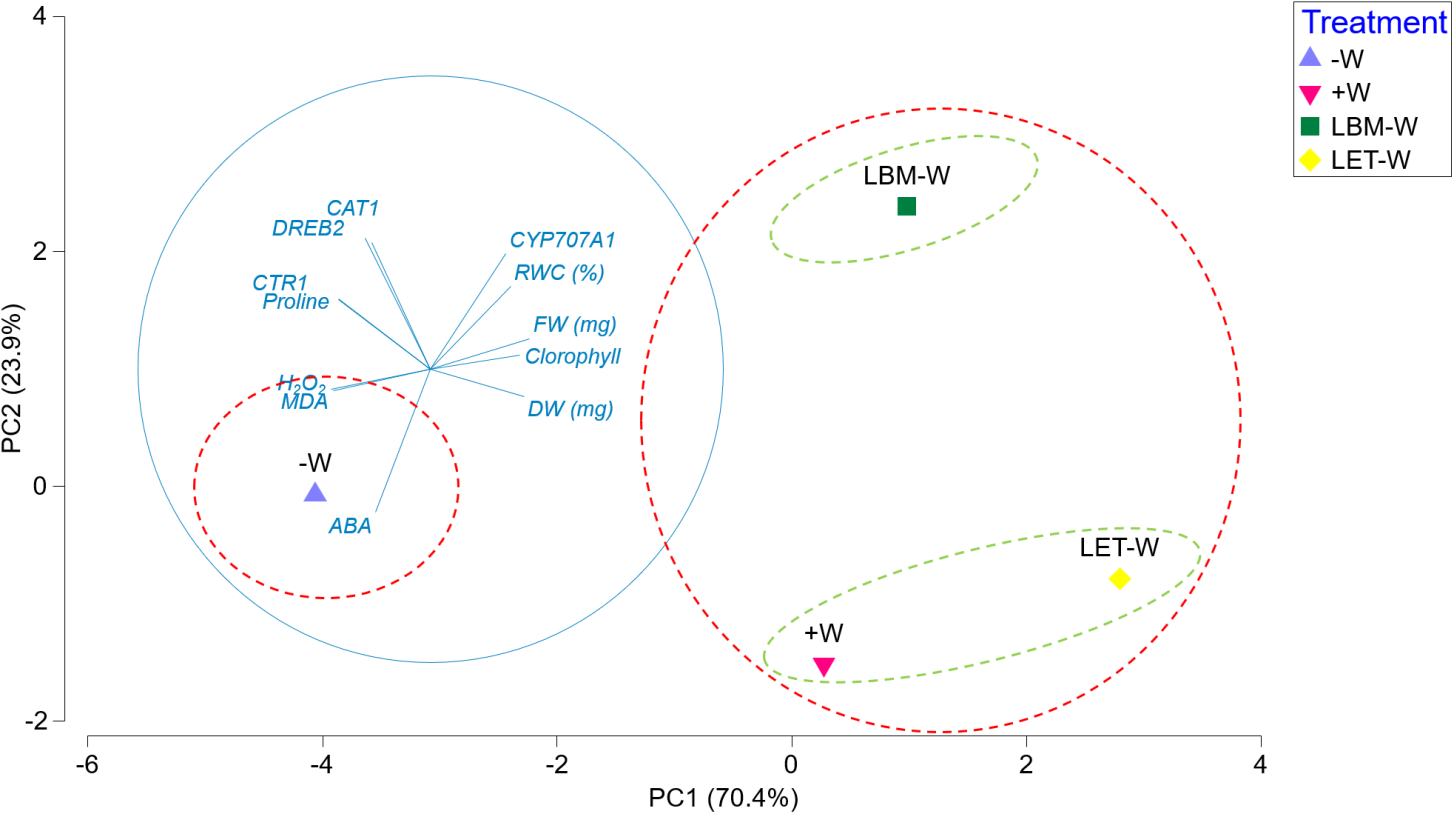
Principal component analysis plot derived from the means of all plant growth, physiological, and biochemical parameters, as well as gene expression results. Principal component axes 1 and 2 collectively accounted for 94.3% of the total variation present. Major groups are represented by red dashed circles, and minor groups are represented by green dashed circles.

PCA graphically explains the relationship between all analyzed variables in this study and the clustering of the different treatments (**Figure 5**). The first two components explained 94% of the total variability.

PC1, which explained 70.4%, defined two clusters: No. 1 comprised water stressed/non-inoculated plants (-W), and No. 2 consisted of inoculated plants under water deficiency (LBM-W; LET-W) and non-stressed/non-inoculated plants (+W). This grouping was strongly explained by practically all studied traits. FW, DW, chlorophyll content, RWC, and upregulation of the *CYP707A1* gene correlated positively with cluster 2, while MDA, proline, H_2_O_2_, and ABA content, as well as upregulation of *DREB2*, *CAT1*, and *CTR1* genes, correlated positively with cluster 1 (**Figure 5**).

Another two subtle clusters consisted of plants under water deficiency inoculated with LBM (LBM-W) on the one hand, and non-stressed plants (+W) and plants under water deficiency inoculated with LET (LET-W) on the other (**Figure 5**). These groups were explained by PC2 (23.9% of variability). This was mainly attributed to the ABA content, which was statistically lower in +W and LET-W, while the remaining parameters played a lesser role.

## 4 Discussion

The role of rhizobia as a nitrogen fixer in symbiosis with legumes is well-known and documented (Werner, 1992; Spaink, 1995; Franche et al., 2009) and commercial rhizobia inoculants have been successfully used worldwide (Rebah et al., 2007; Naseer et al., 2019). Conversely, even if the use of rhizobia strains as biostimulants in non-leguminous plants is also well known, it has been less studied, and little is known about the action mechanisms (Mehboob et al., 2009). This work deepens the mechanisms by which rhizobia enhance crop productivity in non-legume crops under abiotic stress caused by drought conditions, which is very relevant information in the present climate change situation that negatively influences agricultural production (Kaushal and Wani, 2016; Etesami and Maheshwari, 2018; Gamalero and Glick, 2022).

The PGP effect of the four rhizobia strains included in this study has already been demonstrated in wheat (Mulas, 2015), but the action mechanisms have not been studied thus far. Our results showed a clear effect of the four strains in wheat plant growth promotion, increasing FW between 23 and 80% and DW between 3 and 21%, confirming the results previously obtained by Mulas (2015). Similarly, the PGPR action of rhizobia has been previously reported not only in wheat (Kaci et al., 2005; Yanni et al., 2016), but also in radish (Antoun et al., 1998), rice (Yanni et al., 1997; Biswas et al., 2000), sugarcane (Matos et al., 2021), and cotton (Romero-Perdomo et al., 2021). However, despite the fact that rhizobia are commonly used microbial biostimulants, their role in enhancing drought stress tolerance in non-leguminous areas is lacking (Hussain et al., 2014; Naseem et al., 2018). Therefore, our work is, to the best of our knowledge, novel.

LET and LBM clearly produced a positive plant response under drought conditions that encompassed growth parameters and biochemical and physiological responses. This response was stronger in plants inoculated with the LET strain, whose parameters were similar to those of non-stressed plants (+W). FW, DW, chlorophyll content, and RWC were improved by LBM and LET in water-stressed plants. Chlorophyll content is an indicator of the photosynthetic capability of plants; therefore, a higher content involves better drought tolerance (Palta, 1990). Similarly, relative water content (RWC) is an indicator of plant water status in terms of the physiological consequences of cellular water deficit (Smart and Bingham, 1974). Thus, in our research, chlorophyll content and RWC were positively correlated with a better performance of the plants under drought stress, and thus, it is related to a better stress tolerance, as previously demonstrated by several authors (Gontia-Mishra et al., 2016; Hou et al., 2018; Wang et al., 2018).

In addition to growth promotion, LBM and LET also triggered other biochemical and genetic responses in wheat plants, which demonstrates a drought tolerance response. One of the most common responses to water stress is related to osmotic adjustment and antioxidant defense (Sun et al., 2020). Proline is an amino acid that acts as an osmolyte in saline and drought conditions, and it works as both an osmoprotectant and a stress marker (Kerbab et al., 2021). Plants synthesize proline in response to stress, which triggers reactions such as reactive oxygen species (ROS) detoxification, reduction of lipid peroxidation, and structural adaptation of membranes to improve tolerance (Hare and Cress, 1997; Per et al., 2017; Kumar et al., 2021). In this study, control plants under drought stress showed, as expected, an increase in proline content compared to non-stressed plants, while stressed plants inoculated with rhizobia displayed lower levels of proline, indicating that rhizobia alleviated the stress of plants under water deficiency. However, the response of proline levels in stressed plants inoculated with PGPR is erratic, according to the literature; Kerbab et al. (2021) suggested that proline levels depend on different bacterial species, the mechanism of bacterial communication with the plant, the interaction between bacteria, and the intensity of the stress.

In our results, H_2_O_2_ and MDA content decreased significantly in plant tissues when water-stressed plants were inoculated with rhizobia strains. Similar results have been reported in many works (Wang et al., 2012; Gontia-Mishra et al., 2016; Curá et al., 2017; Kumar et al., 2019). H_2_O_2_ is a ROS produced by plants under salinity and drought stress, causing oxidative stress (Gill and Tuteja, 2010). MDA is a product of fatty acid peroxidation and is considered an indicator of the degree of injury in stressed plants (Morales and Munné-Bosch, 2019). ROS produced under biotic or abiotic stress, damage organic molecules, such as lipids, which increase the MDA content and the permeability of the plasma membrane; subsequently, high levels of MDA entail a major stress condition (Shi et al., 2018; Sun et al., 2020; Zhang et al., 2021). Thus, MDA and H_2_O_2_ content are indicators of oxidative stress in plants, and a reduction of both involves a reduction in stress conditions.

A further step was to analyze gene expression related to plant stress situations. In our work, the expression of the *CAT1* gene was downregulated in stressed plants inoculated with the rhizobia strains compared to non-inoculated plants. The *CAT1* gene encodes a catalase responsible for the elimination of H_2_O_2_ and ascorbate peroxidases (Feirabend, 2005). Therefore, plants activate catalases to minimize oxidative damage as part of their defense response to external stress (Luna et al., 2005). Consequently, the observed downregulation of *CAT1* indicates a reduction in catalase activity, which, together with the reduction in MDA and H_2_O_2_ content, clearly indicates a stress reduction in plants under water shortage conditions that were inoculated with rhizobia. A reduction in catalase activity in stressed plants inoculated with PGPR has previously been reported in wheat (Upadhyay et al., 2012; Gontia-Mishra et al., 2016).

Dehydration-responsive element binding (DREB) is a gene that encodes for a transcription factor (TF) that interacts with promoter regions of genes involved in stress responses, regulating their expression and thus enhancing plant tolerance to environmental stress. Specifically, the subclass *DREB2* is induced by dehydration, such as drought stress, and this induction may be either ABA-dependent or ABA-independent (Agarwal et al., 2017; Dong et al., 2017; Akbudak et al., 2018). In our experiment, the *DREB2* gene was downregulated in non-stressed wheat plants and in drought-stressed wheat plants inoculated with the LET strain. These results suggest that the reduction of water deficiency stress exerted by LET is dependent on *DREB2*. Conversely, in stressed plants inoculated with LBM, the expression of *DREB2* was slightly upregulated, although it was not statistically significant, suggesting that LBM reduces water stress independently from *DREB2*. Moreover, the LBM strain strongly reduced the ABA content in water-stressed plants compared to the positive control, which supports the statement that *DREB2* induction is ABA-independent (Lephatsi et al., 2021). Therefore, our results indicate that the reduction of drought stress by PGPR is not always explained by *DREB2* expression patterns or that the expression is strain-dependent. In agreement with our results, the scarce number of studies on the role of PGPR in *DREB2* expression patterns in drought-stressed plants is contradictory. Gontia-Mishra et al. (2016) reported the downregulation of *DREB2* in wheat plants exposed to drought stress and inoculated with *Klebsiella* sp., *Enterobacter ludwigii*, and *Flavobacterium* sp.; conversely, other authors have reported an upregulation of *DREB2* (Sarma and Saikia, 2014; Barnawal et al., 2017; Vaishnav and Choudhary, 2019).

Constitutive Triple Response (*CTR1*) is a negative regulator of the ethylene response pathway encoding a Raf-like protein kinase. When the receptors perceive ethylene, *CTR1* kinase activity is shut off, thereby leading to the expression of stress symptoms in plants (Zhong and Chang, 2012). In other words, when the plant is under stress, ethylene is produced, and *CTR1* is downregulated. Therefore, compared to the water-stressed control, the upregulation of *CTR1* was expected in the non-stressed control and water-stressed treatments inoculated with LBM and LET because of the stress alleviation exerted by the bacteria. However, unexpectedly, the expression of the *CTR1* gene was very similar in the treatments and controls, with no significant differences; even LBM produced *CTR1* downregulation compared with the non-inoculated water-stressed control. We hypothesized that in our experiment, ethylene levels were not affected by the treatments, and for that reason, the levels of *CTR1* expression were similar in the treatments and controls.

Furthermore, the exposure of wheat plants to drought stress caused ABA accumulation; in contrast, a strong decline in ABA content was observed in stressed plants inoculated with LET and more sharply with LBM. ABA is a stress hormone produced in tissue plants in response to water deficit or salinity conditions; this hormone is rapidly accumulated, inducing, among others, stomatal closure, which avoids water loss by transpiration and plant growth (Zhang et al., 2006; Sun et al., 2020). The reduction of ABA levels in stressed plants inoculated with PGPR has been reported in crops, such as wheat with *Arthrobacter protophormiae* and *Dietzia natronolimnaea* (Barnawal et al., 2017) and cucumber with *Burkholderia cepacia*, *Promicromonospora* spp., and *Acinetobacter calcoaceticus* (Kang et al., 2014). This was supported by cytochrome P450 monooxygenase 707A1 (*CYP707A1*) gene expression. The *CYP707A1* gene encodes the ABA catabolic enzyme, ABA8′-hydroxylase, which plays an important role in controlling ABA content in plant tissue, reducing ABA levels (Kushiro et al., 2004; Okamoto et al., 2006). *CYP707A1* was upregulated in non-water-stressed plants and plants under water deficiency inoculated with bacteria, which agrees with the observed reduction in ABA content.

Some of the typically known plant growth-promoting actions exerted by bacteria could be indirectly related to drought tolerance, according to the literature. Therefore, we hypothesized a relationship between the most outstanding PGP activities of LBM and LET, which were evaluated by Mulas (2015), and the observed improvement of drought tolerance. The most outstanding PGP action of LBM is mineral phosphate solubilization (Mulas, 2015). Although the capacity to solubilize phosphate is a recognized direct mechanism of growth promotion (Glick, 2012), adequate phosphorus nutrition is essential for root development and water absorption (Gutiérrez-Boem and Thomas, 1998; Rouphael et al., 2012; Tariq et al., 2017). Therefore, it has been widely recognized that P improves drought tolerance (Nelsen and Safir, 1982; Jin et al., 2006; Alvarez et al., 2009; Cortina et al., 2013; Abdelmoneim et al., 2014; Tariq et al., 2017). Therefore, solubilizing phosphate rhizobacteria can provide assimilable P to plants and ensure adequate P nutrition, indirectly enabling plants to overcome drought stress situations (Kaushal and Wani, 2016; Etesami and Maheshwari, 2018). Furthermore, some PGPR involved in the alleviation of drought stress include phosphate solubilization among their PGP traits, such as *Mesorhizobium ciceri* in chickpea (Yadav et al., 2021), *Streptomyces laurentii* and *Penicillium* sp. in great millet (Kour et al., 2020), and *Pantoea alhagi* in wheat (Chen et al., 2017). Therefore, wheat phosphorus dynamics in plants inoculated with LBM and exposed to drought stress should be addressed in future assays because LBM induced a very sharp reduction in water stress, as revealed by the reduction of ABA, one of the most relevant indicators of drought stress (Etesami and Maheshwari, 2018; Kumar et al., 2019).

However, the production of phytohormones, especially indoleacetic acid (IAA) by PGPR, increases the tolerance to drought stress in an indirect manner because IAA promotes the growth of shoots and roots by modifying the root architecture and enhancing water and nutrient uptake (Marulanda et al., 2009; Kaushal and Wani, 2016; Etesami and Maheshwari, 2018; Ha‐tran et al., 2021; Gamalero and Glick, 2022). LET stands out due to IAA production (Mulas, 2015). Moreover, LET dramatically improved the root development of wheat plants subjected to drought stress compared to non-inoculated plants (Unpublished data; **Figure 1** and **Supplementary Data**). In addition, IAA produced by PGPR causes ROS detoxification (Etesami and Maheshwari, 2018; Gamalero and Glick, 2022), which agrees with the decrease in MDA and H_2_O_2_ content in wheat plants under water deficiency and inoculated with LET compared to non-inoculated plants.

## Conclusions and future remarks

Two (LET and LBM) of the four *Rhizobium leguminosarum* strains tested in this work alleviated water deficiency stress, as demonstrated by several indicators, namely growth rate, biochemical parameters, and the expression of genes related to water stress tolerance. The values observed for the mentioned indicators in plants under water deficiency and inoculated with LET and LBM were similar to those of non-stressed plants. Conversely, from our results, ethylene metabolism seemed uninvolved in the alleviation of drought stress exerted by the two strains of *R*. *leguminosarum*,* *and further studies should focus on unraveling the role of ethylene in drought tolerance mediated by MPB. This research was conducted with rhizobia because they are well-known MPBs. This work has shed light on the mechanisms mediated by MPB in the development of tolerance to water shortages in plants. Currently, the successful methodology applied to rhizobia is being applied to other MPB belonging to other bacterial genera, such as *Bacillus*, *Pseudomonas *and *Azotobacter*,* *to determine if the same mechanisms are involved in drought tolerance development, which is very important in the present scenario of climate change.

## Data availability statement

The original contributions presented in the study are included in the article/**Supplementary Materials**. Further inquiries can be directed to the corresponding author.

## Author contributions

MB: Investigation, Formal analysis, Conceptualization, Writing - Original Draft; JP: Formal analysis, Writing - Original Draft AL-M: Formal analysis, Writing - Review and Editing; NO-L: Formal analysis; JB: Writing - Review and Editing, Supervision, Funding acquisition, FG-A: Conceptualization, Writing - Review and Editing, Supervision, Project administration. All authors contributed to the article and approved the submitted version.

## Funding

This project has been financially supported by European Commission - BBI JU project “Bio-based FERtilising products as the best practice for agricultural management SusTainability (BFERST)”. H2020-BBI-JTI-2018, Grant agreement ID: 837583. NO-L was granted a fellowship from the FPU program by the Spanish Ministry of Education with code (FPU 17/04201).

## Conflict of interest

Authors AL-M and JB are employed by FERTIBERIA S.A.

The remaining authors declare that the research was conducted in the absence of any commercial or financial relationships that could be construed as a potential conflict of interest.

## Publisher’s note

All claims expressed in this article are solely those of the authors and do not necessarily represent those of their affiliated organizations, or those of the publisher, the editors and the reviewers. Any product that may be evaluated in this article, or claim that may be made by its manufacturer, is not guaranteed or endorsed by the publisher.
