# Supplementary figures and images for: Mechanisms involved in drought stress tolerance triggered by rhizobia strains in wheat
Source: Front Plant Sci. 2022 Nov 10;13:1036973. doi: 10.3389/fpls.2022.1036973 (PMC9686006; doi:10.3389/fpls.2022.1036973)

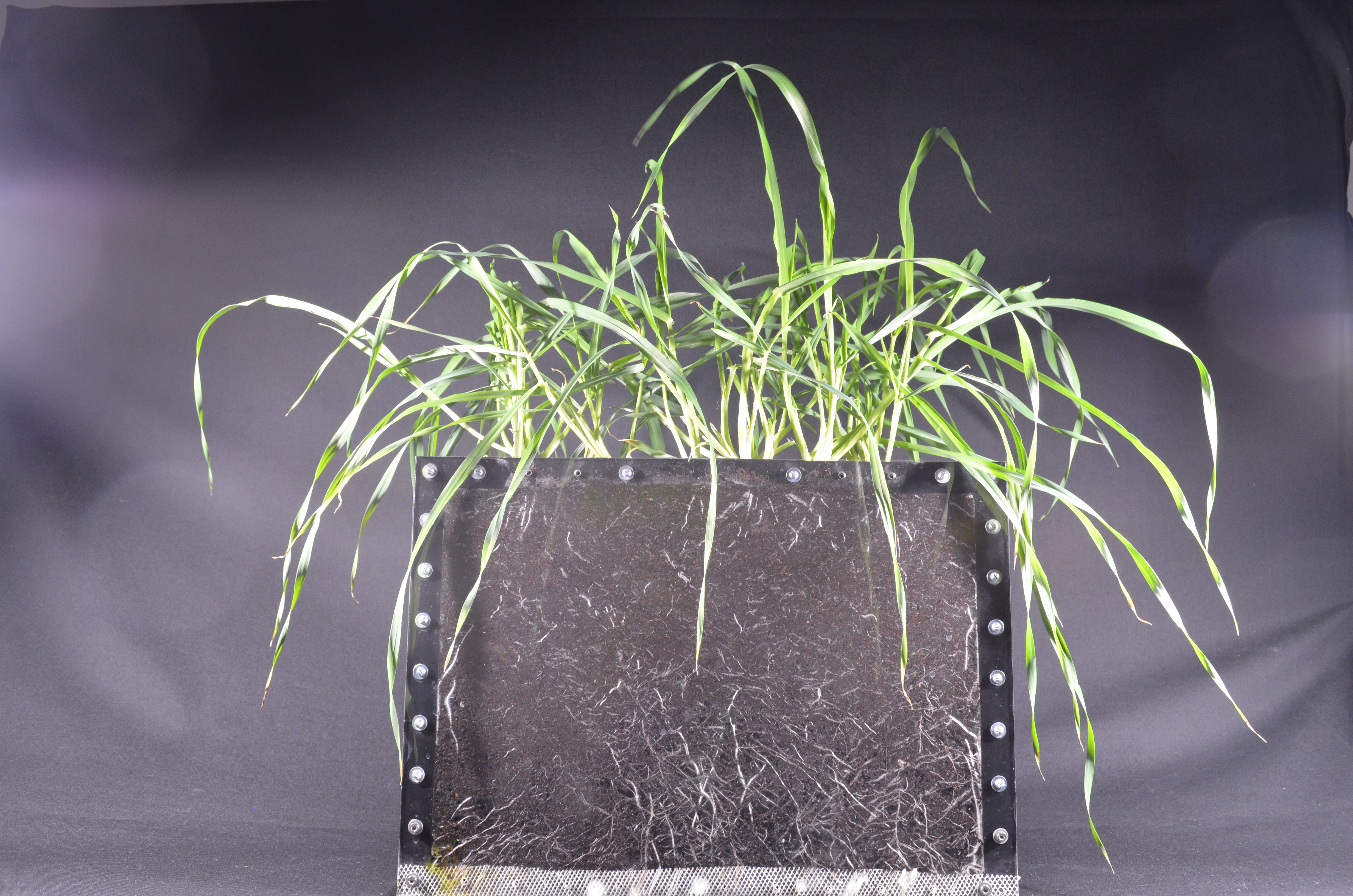

Supplement: Supplementary file 1 [file Image_1.jpg]

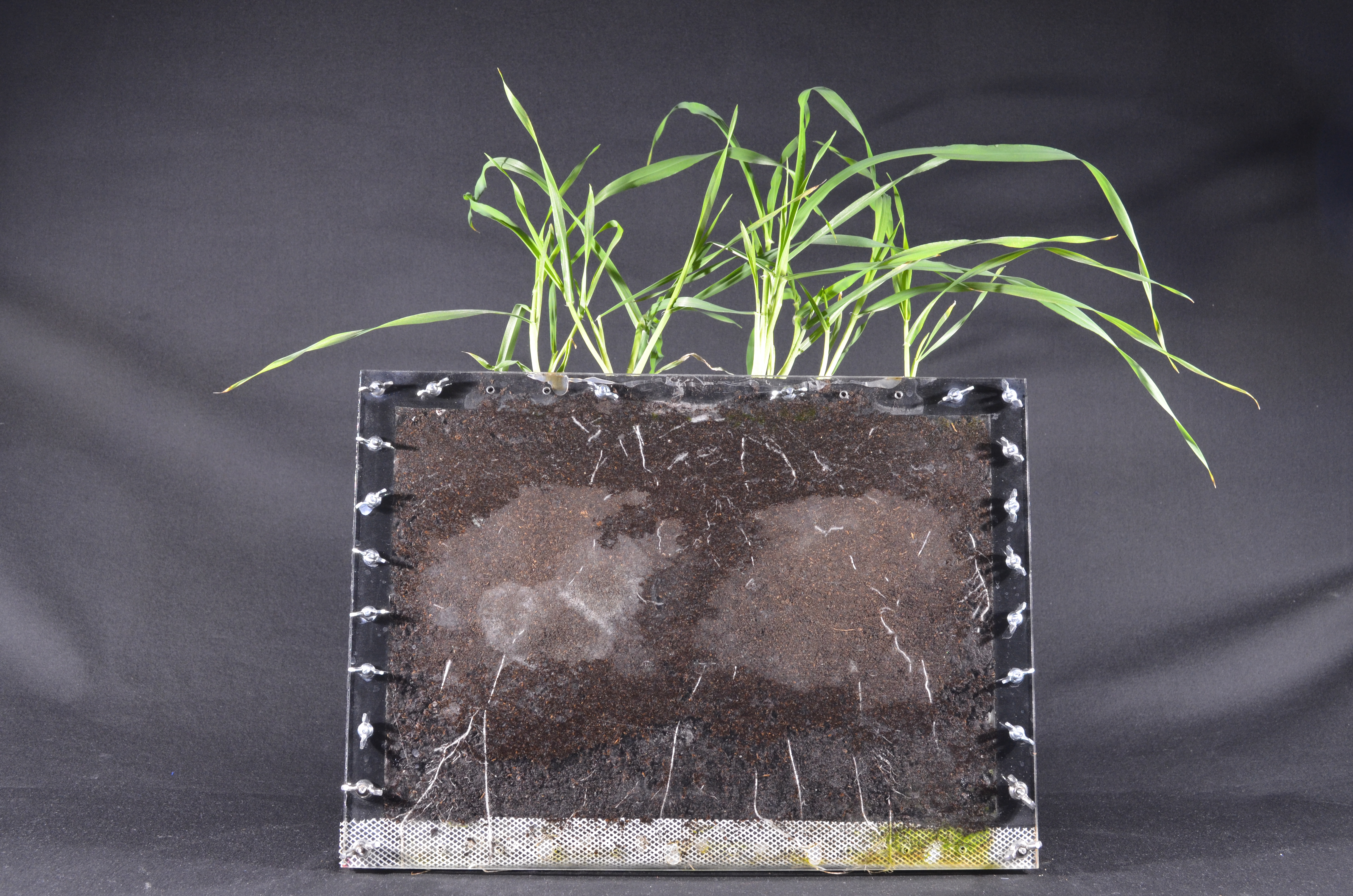

Supplement: Supplementary file 2 [file Image_2.jpg]
