# Supplementary material for: Mechanisms involved in drought stress tolerance triggered by rhizobia strains in wheat
Source: Front Plant Sci. 2022 Nov 10;13:1036973. doi: 10.3389/fpls.2022.1036973 (PMC9686006; doi:10.3389/fpls.2022.1036973)
Supplement: Supplementary file 3 [file Image_3.pdf]

Supplementary material legend

Image 1 are wheat plants under water shortage conditions inoculated with the strain LET1910 whilst Image 2 are uninoculated wheat plants under water shortage
